# Inulin and Fibersol-2 Combined Have Hypolipidemic Effects on High Cholesterol Diet-Induced Hyperlipidemia in Hamsters

**DOI:** 10.3390/molecules21030313

**Published:** 2016-03-05

**Authors:** Wen-Ching Huang, Che-Li Lin, Yi-Ju Hsu, Yen-Shuo Chiu, Yi-Ming Chen, Ming-Fang Wu, Chi-Chang Huang, Ming-Fu Wang

**Affiliations:** 1 Graduate Institute of Sports Science, National Taiwan Sport University, Taoyuan City 33301, Taiwan; magicpica521@gmail.com (W.-C.H.); 1031303@ntsu.edu.tw (C.-L.L.); 1041302@ntsu.edu.tw (Y.-J.H.); 1021301@ntsu.edu.tw (Y.-S.C.); 1021302@ntsu.edu.tw (Y.-M.C.); john5523@ntsu.edu.tw (C.-C.H.); 2 Department of Orthopedic Surgery, Shuang Ho Hospital, Taipei Medical University, New Taipei City 23561, Taiwan; 3 School of Nutrition and Health Sciences, Taipei Medical University, Taipei City 11031, Taiwan; 4 Z-Plus Biotechnology Co., Ltd., Hami St., Datong Dist., Taipei City 10367, Taiwan; minfang58@gmail.com; 5 Department of Food and Nutrition, Providence University, Taichung City 43301, Taiwan

**Keywords:** hypolipidemic, inulin, resistant maltodextrin, high-cholesterol diet, lipid-profiles

## Abstract

The resistant carbohydrates, inulin, and Fibersol-2, belong to soluble dietary fibers and are considered important prebiotics that maintain biological functions, including glucose homeostasis, lipid regulation, colon disease prevention, and prebiotics characteristics. However, few studies have investigated Fibersol-2 alone or in combination with inulin to assess a pooled effect on modulation of hyperlipidemia. We aimed to investigate the effects of this combined supplement (defined as InF) on hamsters fed a 0.2% cholesterol and 10% lard diet (*i.e.*, high-cholesterol diet, HCD) to induce hyperlipidemia. A total of 40 male hamsters were randomly assigned to five groups (*n* = 8 per group) for treatment: standard diet, vehicle (control); or vehicle or InF supplementation by oral gavage at 0, 864, 1727, or 2591 mg/kg/day for eight weeks, designated HCD, InF-1X, InF-2X, and InF-3X groups, respectively. The hypolipidemic efficacy and safety of InF supplementation was assessed by serum lipid indexes, hepatic and fecal lipid content, and histology. InF supplementation significantly improved serum levels of triacylglycerol (TG) and low-density lipoprotein cholesterol (LDL-C) and the ratio of LDL-C/HDL-C after two-week treatment, and reduced serum total cholesterol (TC) levels after four-week administration. After eight-week supplementation, InF supplementation dose-dependently improved serum levels of TC, TG, HDL-C, and LDL-C; LDL-C/HDL-C ratio; and hepatic TC and TG levels. It inhibited TC absorption by feces elimination. Our study provides experiment-based evidence to support that this prebiotics remedy may be useful in preventing or treating hyperlipidemia.

## 1. Introduction

According to the global epidemiological statistics, non-communicable diseases (NCDs) are the main factor affecting socio-economic development and human health [1]. The 66th World Health Assembly (WHA), held in Geneva, Switzerland, endorsed the Global Action Plan for the Prevention and Control of NCDs 2013–2020 (resolution WHA66.10). Therefore, NCDs are a major challenge to public health and affect social and economic development worldwide. The action plan focused on the NCDs cardiovascular disease, cancer, chronic respiratory diseases, and diabetes, with the highest morbidity and mortality. The economic costs of NCDs in low- and middle-income countries is about 7.28 trillion US dollars, with cardiovascular disease accounting for more than 50% of the cost [1]. A retrospective study reported that the economic burden of cardiovascular events with hyperlipidemia is substantial up to three years and increases with non-inpatient use [2].

Hyperlipidemia is a risk factor and highly associated with cardiovascular diseases [3]. Anti-hyperlipidemia was investigated in different aspects, including pharmaceutical mechanisms [4], nature products [5], exercise fitness [6], and gut flora [7]. A hyperlipidemia model with high similarity to human lipid metabolism is important to study. Previous studies have shown that hamsters are similar to human beings in lipid metabolism (e.g., the synthesis and secretion of cholesterol), whereas in models of rat, mouse, pigeon, and quail, lipoprotein metabolism differs from that in humans [8]. Another report also showed that hamsters might be a good animal model for hypercholesterolemia because the content of hyperlipidemia was easily maintained with high-fat, high-cholesterol-diet induction [9]. Therefore, hamsters are widely used to investigate hyperlipidemia [10,11].

Inulin is soluble dietary fiber composed of several simple sugars such as oligosaccharides, belonging to a group of carbohydrates known as fructans, which are indigestible for humans. Inulin has been studied for several physiological functions, including hepatic lipid synthesis gene regulation, increased muscular lipoprotein lipase, beneficial gut-flora fermented products, prebiotics characteristics, and lipid and glucose homeostasis [12,13,14,15]. Fibersol-2 was a patented resistant maltodextrin and produced from corn starch by pyrolysis and enzymatic treatment with random 1,2-, 1,3- α or β glucose linkages [16]. Fibersol-2 was found to have important physiological functions including glucose homeostasis [17], hyperlipidemia regulation [18], prevention of colon disease [19], and prebiotics characteristics. Fibersol-2 was generally recognized as safe by the US Food and Drug Administration (FDA) as maltodextrin and is used in different commercial products.

The lipid regulations of inulin and Fibersol-2 have been reported; however, we have limited research of the effects of such prebiotics over time and the hepatic pathology. Therefore, we aimed to evaluate the inulin and Fibersol-2 combination for lipid-lowering effects in a hamster animal model.

## 2. Results

### 2.1. Growth Curve and Daily Consumption

**Figure 1:**
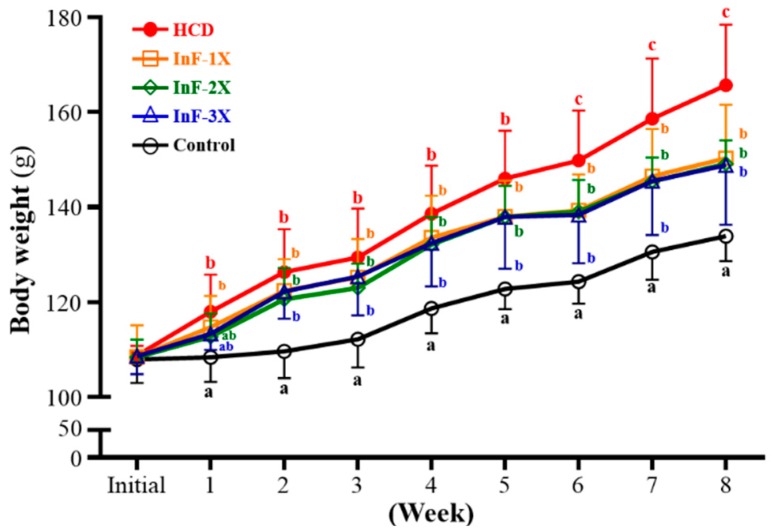
Body weight during the experiment. After one-week adaption, animals were fed a standard laboratory diet or an HCD and the indicated InF supplementation (8 hamsters/each group). Data are mean ± SD; values at the same time with different letters (a, b, c) differ significantly at *p* < 0.05 by one-way ANOVA.

The growth curve for hamsters is shown in Figure 1. At the adaption phase, the initial mean BW was about 108 g in the 5 groups (*p* > 0.05). After 1-week HCD induction, the HCD and InF-1X groups were significantly heavier than controls (*p* < 0.05). The BW of all induction groups significantly increased up to week six as compared with controls (*p* < 0.05). The InF-1X, InF-2X, and InF-3X groups showed significantly ameliorated weight gain after five-week supplementation, but BW was still higher than for controls (*p* < 0.05). The increase in BW was stable and steady in each group. Therefore, InF supplementation could significantly control the abnormal growth induced by HCD. The daily diet and water consumption is shown in Table 1. BW did not differ among supplementation groups (*p* > 0.05).

### 2.2. Effect of InF Supplementation on Body Composition at the End of the Experiment

At eight weeks after InF supplementation, liver, kidney, heart, lung, and EFP were carefully excised, collected, and weighed for evaluation of body composition. Liver, kidney, heart, lung, and EPF absolute weights but not relative heart and lung relative (%) weight differed among groups (Table 2). The liver and EFP weight were higher, by 1.78- and 1.58-fold (*p* < 0.0001), for the HCD than control group. The liver weight was lower, by 13.52% (*p* = 0.001), 18.1% (*p* < 0.0001) and 18.65% (*p* < 0.0001), with InF-1X, InF-2X, and InF-3X treatment, respectively, than HCD alone. The EFP weight was lower by 11.88% (*p* = 0.0005), 21.75% (*p* < 0.0001), and 22.69% (*p* < 0.0001), respectively. Liver and EPF relative weight (%) showed similar findings.

### 2.3. Effect of Two-Week InF Supplementation on Serum Lipid Profiles in Hyperlipidemic Hamsters

**Figure 2:**
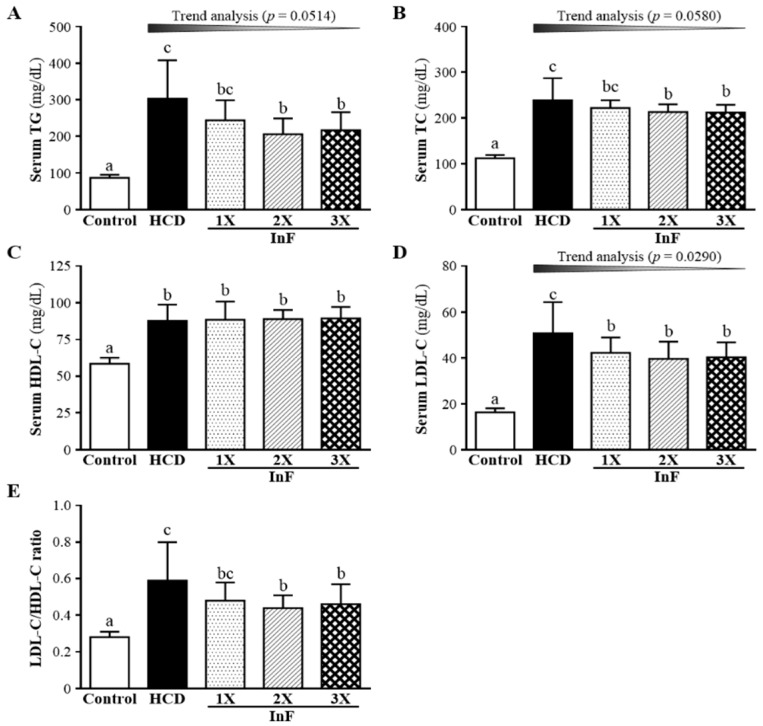
Effect of two-week InF supplementation on serum TG (**A**); TC (**B**); HDL-C (**C**); LDL-C (**D**) levels; and LDL-C/HDL-C ratio (**E**) in hyperlipidemic hamsters. Data are mean ± SD (*n* = 8/group); values with different letters (a, b, c) differ significantly at *p* < 0.05 by one-way ANOVA.

At two weeks after InF supplementation, all lipid levels were higher with HCD alone than controls. Serum TG level was lower, by 31.94% (*p* = 0.003) and 28.22% (*p* = 0.008), with 2X and 3X InF supplementation than HCD alone. TC level did not differ between InF supplementation and HCD alone (*p* > 0.05) (Figure 2B), nor did HDL-C level (Figure 2C). LDL-C level was significantly lower with InF supplementation than HCD alone (Figure 2D), as was LDL-C/HDL-C ratio (Figure 2E).

### 2.4. Effect of Four-Week InF Supplementation on Serum Lipid Profiles in Hyperlipidemic Hamsters

**Figure 3:**
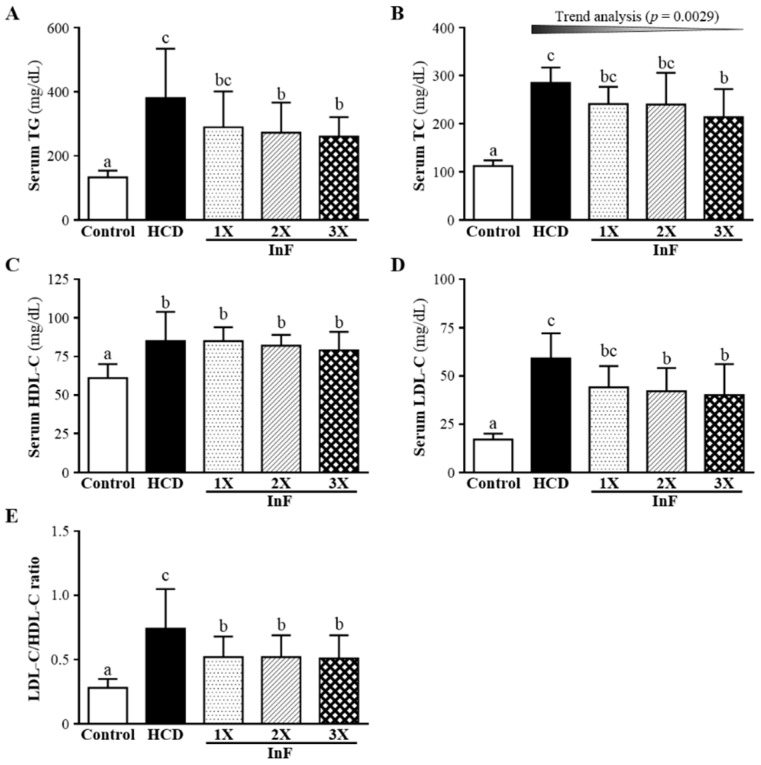
Effect of four-week InF supplementation on serum TC (**A**); TG (**B**); HDL-C (**C**); LDL-C (**D**) levels; and LDL-C/HDL-C ratio (**E**) in hyperlipidemic hamsters. Data are mean ± SD (*n* = 8/group); values with different letters (a, b, c) differ significantly at *p* < 0.05 by one-way ANOVA.

At 4 weeks after treatment, all lipid levels were higher with HCD alone than control treatment (Figure 3). Only HDL-C level showed no difference with InF treatment than HCD alone. For TG, TC, LDL-C and LDL-C/HDL-C ratio, levels were lower with InF treatment than HCD alone.

### 2.5. Effect of Eigh-Week InF Supplementation on Serum Lipid Profiles in Hyperlipidemic Hamsters

**Figure 4:**
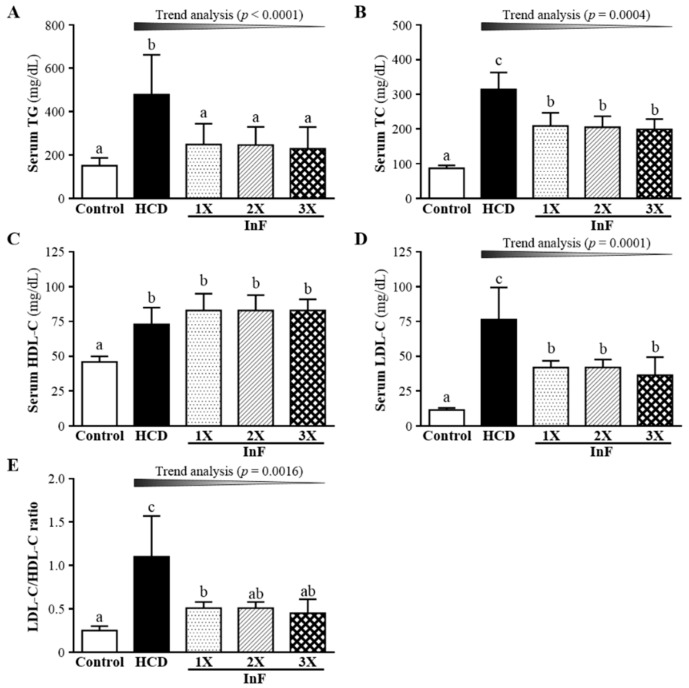
Effect of eight-week InF supplementation on serum TC (**A**); TG (**B**); HDL-C (**C**); LDL-C (**D**) levels; and LDL-C/HDL-C ratio (**E**) in hyperlipidemic hamsters. Data are mean ± SD (*n* = 8/group); values with different letters (a, b, c) differ significantly at *p* < 0.05 by one-way ANOVA.

At 8 weeks after treatment, all lipid levels were higher with HCD alone than control treatment (Figure 4). Only HDL-C level showed a difference with InF treatment than HCD alone. HDL-C level was higher with InF supplementation than HCD alone. For TG, TC, LDL-C and LDL-C/HDL-C ratio, levels were lower with InF treatment than HCD alone.

### 2.6. Effect of Eight-Week InF Supplementation on Hepatic TG and TC Levels in Hyperlipidemic Hamsters

**Figure 5:**
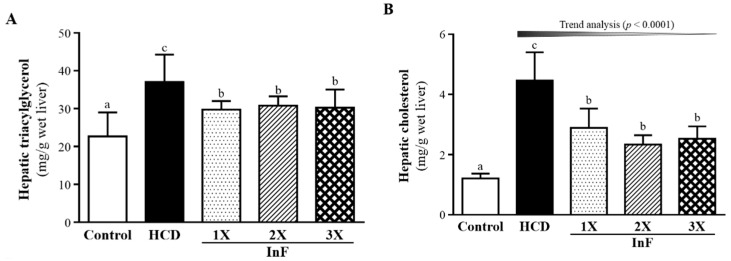
Effect of eight-week InF supplementation on hepatic TG (**A**) and TC (**B**) levels in hyperlipidemic hamsters. Data are mean ± SD (*n* = 8/group); values with different letters (a, b, c) differ significantly at *p* < 0.05 by one-way ANOVA.

TG and TC content in liver was higher with HCD alone than control treatment and lower with InF treatment than HCD alone (Figure 5A,B). Thus, InF supplementation could significantly mitigate the hepatic TC and TG content accumulation induced by HCD hyperlipidemia.

### 2.7. Effect of Eight-Week InF Supplementation on Fecal TG and TC Levels in Hyperlipidemic Hamsters

**Figure 6:**
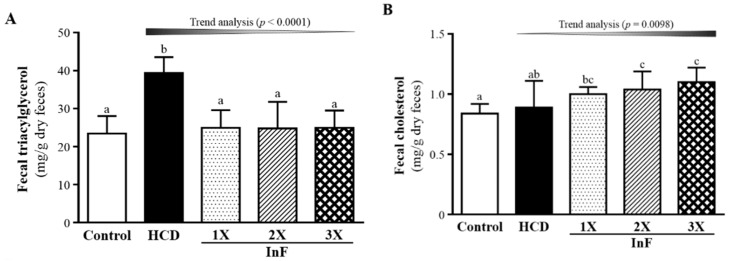
Effect of eight-week InF supplementation on fecal TG (**A**) and TC (**B**) levels in hyperlipidemic hamsters. Data are mean ± SD (*n* = 8/group); values with different letters (a, b, c) differ significantly at *p* < 0.05 by one-way ANOVA.

Fecal TG content was higher with HCD than control treatment (Figure 6A) and TG content was lower with InF supplementation than HCD alone. Fecal TC content was higher with HCD than control treatment (Figure 6B). However, TC content dose-dependently increased with InF supplementation as compared with HCD alone. Therefore, excess cholesterol could be expelled into feces after daily HCD diet intake with InF supplementation.

### 2.8. Effect of InF Supplementation on Tissue Features

**Figure 7:**
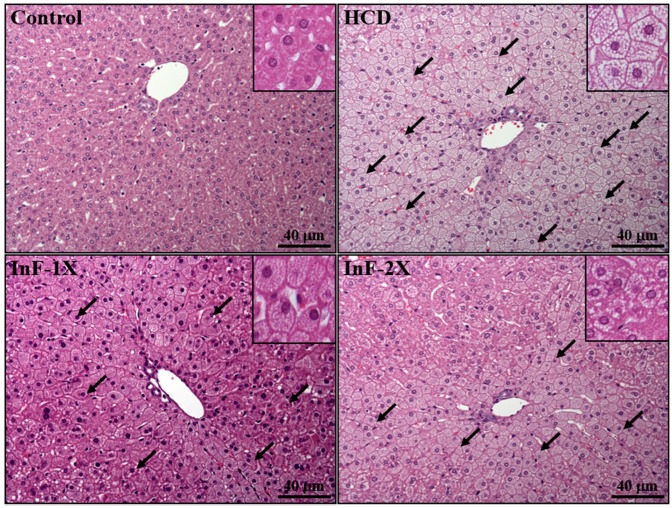

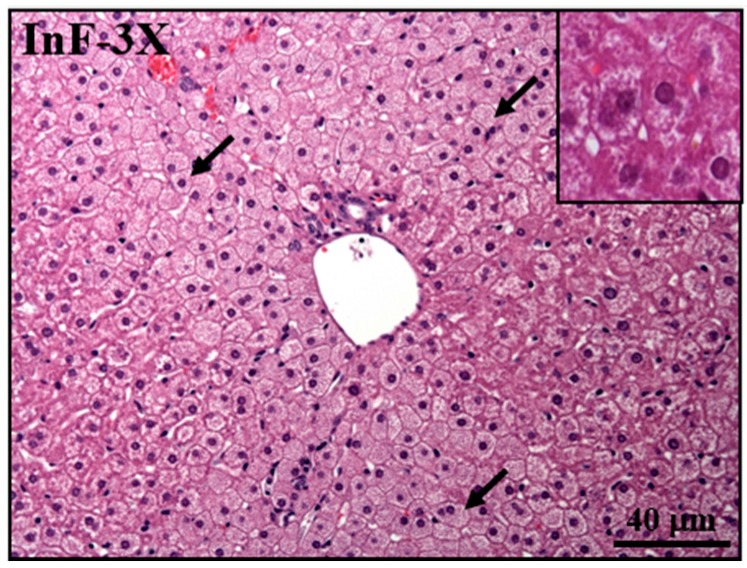
Effect of eight-week InF supplementation on the pathology of liver tissues in hyperlipidemic hamsters. Arrows indicate microvesicular fat droplets. Specimens were photographed by light microscopy. (H and E staining, magnification: ×200, Scale bar, 40 μm).

At eight weeks of InF supplementation, the liver usually exhibited clear pathological characteristics in hamsters with hyperlipidemia. Controls showed a clear hepatic cord and sinusoid (Figure 7). However, the morphology of steatosis differs among rodent species. Steatosis is mixed (macro- and microvesicular) in mice [20] and is microvesicular in hamsters. HCD hamsters showed fatty liver changes, with hepatocytes comprising microvesicles filled with small lipid droplets, similar to previous pathological observations. After InF supplementation, the degree of steatosis was significantly ameliorated as compared with HCD alone. As showed in Figure 7, the diffuse type of microvesicular steatosis was found in the photography in HCD group. However, the number of hepatocytes with small lipid droplets tended to decrease with dose depend in both InF-2X and InF-3X group. The arrows indicated the steatosis areas with significant difference amoung groups.

## 3. Discussion

The remedy of prebiotics, inulin and Fibersol-2, used in this study could significantly ameliorate the hyperlipidemia with time- and dose-dependent manner and exclude the excessive cholesterol intake form HCD diet. The hepatic steatosis was mitigated and the cardiovascular risk was also decreased by long term supplementation with InF.

Prebiotics, first mentioned by Gibson *et al.*, in 1995 [21], are substances, such as indigestible carbohydrates, fermented by microflora, that affect growth, composition, and activity of beneficial microflora in the gastrointestinal tract for health promotion [22]. Prebiotics include fructooligosaccharides, oligofructose, lactulose, and galactooligosaccharides, polysaccharides (starch, resistant starch, and modified starch). The materials investigated in this study, inulin and Fibersol-2, belong to those resistant carbohydrates. In a previous study, synbiotics (prebiotics and probiotics combined) had synergistic effects because they promoted existing beneficial bacteria growth and improved the implantation of newly additive probiotic strains in the colon [21]. Synbiotics strategies were also reported to benefit health in children [23], increase anti-oxidation [24], and regulate lipids [25]. In the current study, we used two potential prebiotics for hyperlipidemia evaluation. Supplementation with inulin and Fibersol-2 combined with hamsters with hyperlipidemia induced by an HCD significantly ameliorated serum levels of TG and LDL-C, the ratio of LDL-C/HDL-C after two-week treatment, and reduced serum TC level after four-week administration. After eight-week supplementation, InF supplementation dose-dependently improved serum levels of TC, TG, HDL-C, and LDL-C and LDL-C/HDL-C ratio, as well as hepatic TC and TG levels, and inhibited TC absorption by feces elimination. This prebiotics remedy, InF, may be useful in preventing or treating hyperlipidemia.

The synbiotics inulin and *Lactobacillus gasseri* were found to decrease plasma TC and LDL-C levels, by 7.84% and 9.27%, respectively, as compared with controls over 12 weeks [26]. Another inulin-related experiment also showed that six-week supplementation could significantly mitigate increased TC and LDL-C levels by 8.7% and 14.4%, respectively [27]. We found significantly improved TG and LDL-C levels with two-week supplementation (Figure 2), improved TG, TC, HDL-C, and LDL-C levels with four-week supplementation (Figure 3) and improved TG, TC, HDL-C, and LDL-C levels with eight-week supplementation (Figure 4). Our combination of two prebiotics could confer effective hypolipidemia with time.

Previous studies demonstrated a variety of inulin doses for hypolipidemic effects. One clinical study reported that 10 g/day inulin for six weeks could significantly decrease TG and hepatic lipogenesis [28] and another study showed significantly reduced serum TG level with 20-g/day inulin supplementation [29]. Adverse gastrointestinal side effects were found with ≥ 30 g/day equivalent doses converted to the effective dose of the animal study. In our effective dose of InF-1X, the inulin was only 2.1 g/day of the human equivalent dose, which should be much lower than that used in previous studies. We also showed that Fibersol-2 could improve hypolipidemic effects.

The short chain fatty acids (SCFAs) propionate, butyrate, and acetate are the major end products of indigestible carbohydrates fermented by microbiota in the upper gastrointestinal tract, and the proportions of SCFA vary depending on indigestible carbohydrates and microorganism species [30]. In previous reports, SCFAs had effects on physiological activities in terms of lipogenesis downregulation [31] and increased lipid catabolism [32] and benefitted dominant microbiota [33]. The significant decrease in hepatic lipid contents we found (Figure 5) could be the physiological activities of SCFAs fermented by microbiota. Fat composition was also studied for the effects of synbiotics supplementation. *Clostridium butyricum* combined with inulin could significantly decrease the epididymis white adipocyte tissue composition and improve TG content [34]. The effects of two prebiotics on fat composition were consistent with our findings of tissue weight with InF supplementation (Table 2).

In terms of feces lipid content, inulin-type fructans do not seem to be able to bind the bile acids (cholesterol conjugated synthesis) present in the intestinal lumen. However, the organic acids produced by fermented indigestible fructans could reduce the environmental pH in the intestinal lumen. Thus, bile acids could be eliminated with feces if less soluble. Increased bile acids synthesis may have contributed to the decreased serum and liver cholesterol expelled by feces. Additionally, butyrate, a SCFA, produced by the fermentation process promotes the thickness of the intestinal wall, which hinders the absorption of cholesterol molecules from the diet [35]. We found excess cholesterol from the HCD diet significantly expelled into feces (Figure 6B), which is consistent with previous mechanisms. In a previous study, the gut microbiota play important roles on modulation of host energy and lipid metabolism via comparison of conventionally raised and germ-free mice. It showed that lipid absorption is not reduced in conventional mice, but the lipid clearance is increased via suppression of angiopoietin-like protein 4/fasting-induced adipose factor (Angptl4/Fiaf) by microbiota regulation [36,37]. In the current study, the microbiota by InF supplementation could be a benefit to lipid clearance and the lipid content in feces was significantly lower than HCD group.

## 4. Materials and Methods

### 4.1. Materials

InF was composed of Fibersol-2 and inulin at about a 7:3 ratio (Funcare of Taiwan, Taichung, Taiwan). Nutrition components included 1 g carbohydrates, 5 g dietary fiber, 0.04 mg sodium without protein and fat content, for 4 kcal per serving (7 g) recommended for daily intake. The manufacturing process and quality control were certified by HACCP and ISO22000.

### 4.2. Animals and Experimental Design

Male hamsters (eight weeks old, SPF grade) were obtained from National Laboratory Animal Center (AAALAC certification) and housed in the animal room at National Taiwan Sport University (NTSU) with temperature (24 ± 1 °C) and humidity (50%–60%). The photoperiod was regularly controlled for a 12-h light-dark cycle (light on 7:00 AM). Hamsters were provided standard laboratory chow diet (No. 5001; PMI Nutrition International, Brentwood, MO, USA) and distilled water *ad libitum*. Before experiments, hamsters were quarantined and acclimated for one week to the environment and diet. All animal experiments were reviewed by the Institutional Animal Care and Use Committee (IACUC) of NTSU for animal welfare care and conformed to the protocol IACUC-10403 approved by the IACUC ethics committee.

The human-equivalent dose conversion was based on a formula from the US FDA by body surface area. The recommended dose as described above was 7 g/day. The dose for humans is about 116.7 mg/kg body weight (BW; for 60-kg adult body weight), and the conversion coefficient 7.4 was used to account for differences in body surface area between hamsters and humans according to US FDA guidelines for estimating the maximum safe starting dose in initial clinical trials of adult healthy volunteers. Therefore, the equivalent dose for hamsters for 1× InF should be 864 mg/kg BW (116.7 × 7.4 = 864). The 2× and 3× InF doses should be 1727 and 2591 mg/kg BW, respectively. All indicated treatments were administered by oral gavage with equal volume (1 mL/kg BW).

After one-week adaption, 40 hamsters were randomly divided into five groups for treatment: normal (*n* = 8), standard chow diet (control), and four groups (*n* = 8/group) fed a high-cholesterol diet (HCD), HCD with 1× InF (InF-1X), HCD with 2× InF (InF-2X), and HCD with 3× InF (InF-3X). Each group (*n* = 8) contained two cages (4 hamster/cage) for animal welfare consideration. The administration volume was the same (0.1 mL/kg BW) for all groups. Food intake, water consumption, and BW were monitored and recorded daily or weekly.

### 4.3. HCD Composition

The preparation and composition of HCD diet was as we previously described [38]. The #5001 chow diet contained 28.5% protein, 13.5% fat and 58.0% carbohydrates, for 3.35 kcal/g. The chow diet was supplemented with 0.2% (*wt*/*wt*) cholesterol and 10% (*wt*/*wt*) lard (both Sigma-Aldrich, St. Louis, MO, USA) as an HCD diet, for 3.92 kcal/g, with 21.96% protein, 33.37% fat and 44.67% carbohydrates.

### 4.4. Liver and Feces Lipid Analysis

Total cholesterol (TC) and triglycerides (TG) measurement was as we previously described [39] with modification. The 20-mg liver tissue was homogenized with 200 μL organic solvent (chloroform : isopropanol: NP40 = 7:11:0.1) and centrifuged at 12,000 *g* for 10 min for the same 100-μL supernatant collections. After evaporation, the dilute buffer (1 M potassium phosphate, pH 7.4, 500 mM sodium chloride, 50 mM cholic acid) was added for dissolving by vortexing and sonication. TC and TG levels were assessed by use of the Cayman Cholesterol Fluorometric and Triglyceride Colorimetric Assay kits (Cayman, MI, USA). Measurement of feces content followed similar procedures, but the extraction was with organic solvent (chloroform:methanol = 2:1, *v*/*v*) and dissolved in DMSO for the same kit analysis.

### 4.5. Clinical Biochemical Profiles

After eight-week treatment, all hamsters were euthanized by 95% CO_2_ asphyxiation after 12-h fasting, and blood was immediately sampled by cardiac puncture. TC, TG, low-density lipoprotein cholesterol (LDL-C), and high-density lipoprotein cholesterol (HDL-C) were measured at weeks two, four, and eight by using the Beckman DxC 800 autoanalyzer (Beckman Coulter, Brea, CA, USA).

### 4.6. Body Composition and Histology of Liver

After sacrifice, the important tissues or organs, including liver, kidney, heart, lung, and epididymal fat pad (EFP), were carefully removed, weighed, and pre-treated in 10% formalin. Liver tissues were carefully removed, minced, and fixed in 10% formalin after sacrifice. All samples were then embedded in paraffin and cut into 4-μm thick slices for morphological and pathological evaluation. Tissue sections were stained with hematoxylin and eosin and examined by using a light microscope equipped with a CCD camera (BX-51, Olympus, Tokyo, Japan) by a veterinary pathologist.

### 4.7. Statistical Analysis

All data are represented as mean ± SD for *n* = 8 hamsters per group. Statistical differences were analyzed by one-way ANOVA and the Cochran–Armitage test for trend analysis of the dose-effect of InF supplementation with use of SAS 9.0 (SAS Inst., Cary, NC, USA). *p* < 0.05 was considered statistically significant.

## 5. Conclusions 

In the current study, we provide information on lipid profiles with prebiotics supplementation to reveal the potential physiological activities under a hyperlipidemic hamster model. The ratio of LDL-C/HDL-C is a reliable predictor of cardiovascular risk. Risk of cardiovascular events is increased with low HDL-C level in patients with coronary artery disease [40]. After eight-week supplementation with prebiotics in our model, the LDL-C/HDL-C ratio could be significantly lowered with prebiotics supplementation as compared with HCD alone, so the treatment may decrease the cardiovascular potential risk. The two prebiotics combined could be effective for lipid regulation with reasonable gastrointestinal tolerance. Such nutritional supplementation could improve health for people with hyperlipidemia. 

## Figures and Tables

**Table 1:** General characteristics of the experimental groups.

Characteristics	Control	HCD	InF-1X	InF-2X	InF-3X
Initial BW (g)	107.9 ± 4.9	108.8 ± 2.0	108.5 ± 6.6	108.4 ± 3.7	108.6 ± 3.8
Final BW (g)	133.9 ± 5.3 ^a^	165.7 ± 12.7 ^c^	150.3 ± 11.3 ^b^	149.1 ± 5.0 ^b^	148.8 ± 12.5 ^b^
Diet (g/hamster/day)	21.3 ± 2.0 ^b^	19.4 ± 2.2 ^a^	19.3 ± 1.9 ^a^	19.3 ± 1.9 ^a^	19.3 ± 2.1 ^a^
Water (mL/hamster/day)	11.0 ± 3.2 ^b^	8.2 ± 1.6 ^a^	8.2 ± 1.8 ^a^	8.2 ± 1.5 ^a^	8.2 ± 1.4 ^a^

Data are mean ± SD (*n* = 8 hamsters/group); values at the same time with different letters (a, b, c) differ significantly at *p* < 0.05 by one-way ANOVA.

**Table 2:** The effects of InF supplementation on body composition.

Characteristics	Control	HCD	InF-1X	InF-2X	InF-3X
Tissue weight (g)					
Liver	3.69 ± 0.32 ^a^	6.58 ± 0.71 ^c^	5.69 ± 0.69 ^b^	5.39 ± 0.11 ^b^	5.36 ± 0.37 ^b^
Kidney	0.97 ± 0.06 ^a^	1.04 ± 0.08 ^b^	0.97 ± 0.07 ^a^	0.94 ± 0.05 ^a^	0.95 ± 0.06 ^a^
Heart	0.46 ± 0.04 ^a^	0.57 ± 0.03 ^c^	0.52 ± 0.06 ^b^	0.52 ± 0.03 ^b^	0.52 ± 0.04 ^b^
Lung	0.77 ± 0.12 ^a^	1.07 ± 0.35 ^b^	0.78 ± 0.06 ^a^	0.78 ± 0.04 ^a^	0.78 ± 0.05 ^a^
EFP	2.51 ± 0.35 ^a^	3.97 ± 0.40 ^c^	3.22 ± 0.54 ^b^	3.10 ± 0.25 ^b^	3.07 ± 0.35 ^b^
Relative tissue weight (%)					
Liver	2.86 ± 0.18 ^a^	4.08 ± 0.27 ^c^	3.91 ± 0.22 ^b,c^	3.74 ± 0.11 ^b^	3.78 ± 0.15 ^b^
Kidney	0.75 ± 0.05 ^b^	0.65 ± 0.05 ^a^	0.67 ± 0.02 ^a^	0.65 ± 0.04 ^a^	0.67 ± 0.04 ^a^
Heart	0.36 ± 0.03 ^a^	0.35 ± 0.03 ^a^	0.36 ± 0.05 ^a^	0.36 ± 0.02 ^a^	0.36 ± 0.03 ^a^
Lung	0.60 ± 0.08 ^a,b^	0.67 ± 0.22 ^b^	0.54 ± 0.03 ^a^	0.54 ± 0.04 ^a^	0.55 ± 0.05 ^a^
EFP	1.95 ± 0.25 ^a^	2.46 ± 0.19 ^c^	2.21 ± 0.27 ^b^	2.15 ± 0.14 ^a,b^	2.16 ± 0.20 ^a,b^

Data are mean ± SD (*n* = 8/group); values with different letters (a, b, c) differ significantly at *p* < 0.05 by one-way ANOVA. EFP, epididymal fat pad.
